# Genetic architecture and genomic patterns of gene flow between hybridizing species of *Picea*

**DOI:** 10.1038/hdy.2015.19

**Published:** 2015-03-25

**Authors:** A De La Torre, P K Ingvarsson, S N Aitken

**Affiliations:** 1 Department of Forest and Conservation Sciences, Centre for Forest Conservation Genetics, University of British Columbia, Vancouver, BC, Canada; 2 Department of Ecology and Environmental Science, Umeå University, Umeå, Sweden; 3 Umeå Plant Sciences Centre, Umeå, Sweden

## Abstract

Hybrid zones provide an opportunity to study the effects of selection and gene flow in natural settings. We employed nuclear microsatellites (single sequence repeat (SSR)) and candidate gene single-nucleotide polymorphism markers (SNPs) to characterize the genetic architecture and patterns of interspecific gene flow in the *Picea glauca × P. engelmannii* hybrid zone across a broad latitudinal (40–60 degrees) and elevational (350–3500 m) range in western North America. Our results revealed a wide and complex hybrid zone with broad ancestry levels and low interspecific heterozygosity, shaped by asymmetric advanced-generation introgression, and low reproductive barriers between parental species. The clinal variation based on geographic variables, lack of concordance in clines among loci and the width of the hybrid zone points towards the maintenance of species integrity through environmental selection. Congruency between geographic and genomic clines suggests that loci with narrow clines are under strong selection, favoring either one parental species (directional selection) or their hybrids (overdominance) as a result of strong associations with climatic variables such as precipitation as snow and mean annual temperature. Cline movement due to past demographic events (evidenced by allelic richness and heterozygosity shifts from the average cline center) may explain the asymmetry in introgression and predominance of *P. engelmannii* found in this study. These results provide insights into the genetic architecture and fine-scale patterns of admixture, and identify loci that may be involved in reproductive barriers between the species.

## Introduction

The genetic architecture of hybrid zones provides the key to understand the sequence of genetic changes that accompany or facilitate speciation (Lexer *et al.*, 2005). It provides information about the interplay between gene flow as homogenizing force facilitating the spread of alleles and maintaining species as cohesive units, and natural selection promoting population divergence (Morjan and Rieseberg, 2004). In addition, characterizing genetic architecture allows the study of hybrid zones as evolutionary filters; hybrid zones may allow the spread of advantageous alleles from one species to another; however, alleles that are adaptive in one species or environment and maladaptive in the other can be maintained (Barton and Gale, 1993; Lexer *et al.*, 2005; Abbott *et al.*, 2013). Hybridization also generates new genetic combinations of parental alleles that can be tested by selection. These new genetic recombinants may have advantages over parental species because of transgressive segregation or adaptive introgression, or disadvantages because of negative epistatic interactions (Arnold *et al.*, 2012; Abbott *et al.*, 2013). Natural hybrid zones may also provide important clues for the identification of important adaptive traits in species with wide distributions and long generations such as trees (Lexer *et al.*, 2007; Lindtke *et al.*, 2012).

Cline theory provides a rich and powerful conceptual framework for understanding the balance between selection and gene flow in the maintenance of hybrid zones, based on estimates of cline width and linkage disequilibrium (Barton and Hewitt, 1985; Barton and Gale, 1993; Gay *et al.*, 2008; Abbott *et al.*, 2013). Cline width can be used to identify regions of extensive versus restricted introgression in the genomes of hybridizing species, and genes that may be involved in reproductive isolation. Different alleles may be favored in different environments or genetic backgrounds, and can be maintained by selection in the presence of random mixing (Barton and Gale, 1993; Hancock *et al.*, 2011). These alleles are expected to contribute to reproductive isolation between species and therefore introgress less than neutral markers, resulting in narrow cline widths (Teeter *et al.*, 2008). At other loci, globally advantageous alleles spread faster and farther than neutral alleles from one species to another because of balancing selection, resulting in wide clines, and reduced differentiation between species. A close examination of genetic clines can also improve our understanding of selection across hybrid zones. Bimodal clines (Jiggins and Mallet, 2000) may result from high dispersal and strong selection against heterozygotes, as is characteristic of tension zones. If premating isolation or selection against hybrids is weak or absent, hybrids will form an identifiable group characterized by intermediate allele frequencies and broad genetic variance because of varying levels of introgression. In this case, the distribution would be best described by a trimodal distribution. Finally, with extensive introgression, the distribution tends to a unimodal distribution, characteristic of a hybrid swarm (Gay *et al.*, 2008).

With the advent of genomic tools to analyze large numbers of loci, novel methods of clinal analysis (genomic clines) have been developed to analyze introgression of individual alleles and genotypes against average levels of genome-wide admixture (Gompert and Buerkle, 2009, 2010). The analysis of genomic clines facilitates the study of interspecific heterozygosity (proportion of loci with alleles from both ancestral populations) which, combined with measurements of ancestry, can provide useful information about hybrid advantage or disadvantage, and the strength of reproductive barriers between parental species (Lindtke *et al.*, 2012). Joint consideration of ancestry and interspecific heterozygosity is particularly important in hybrid zones with several generations of admixture, as it allows for the study of continuous distributions of hybrid genotypes without assuming that only one generation of admixture have occurred (Fitzpatrick, 2012). By studying deviations from neutral expectations in genomic clines, it is possible to differentiate among underdominance, dominance and directional selection for individual loci. Directional selection, also known as ‘excess ancestry', coupled with increased differentiation between parental populations, can identify loci involved in reproductive isolation (Gompert *et al.*, 2012). Combining both genomic and geographic clinal analyses provides a powerful approach to characterize the genetic architecture of the hybrid zone and to identify genomic regions that may be involved in reproductive barriers between species.

*Picea glauca* Moench (Voss; white spruce) is a transcontinental, boreal species that occurs naturally in almost all forested regions in Canada, with the exception of the Pacific Coast. It generally occurs at low elevations, although in Alaska it ranges from low elevations to alpine treeline. *P. engelmannii* Parry ex. Engelm (Engelmann spruce) extends from British Columbia and Alberta in the north, to New Mexico and Arizona in the south. Engelmann spruce grows at high elevations and is restricted to cold, humid habitats. It has a low tolerance of high temperatures and summer drought, but high tolerance of short growing seasons and deep snowpacks (De La Torre *et al.*, 2014a). These wind-dispersed, long-lived, tree species hybridize in areas where their ranges overlap, at intermediate environments between white spruce and Engelmann spruce' habitats. Our study uses 10 single sequence repeat (SSR) and 86 single-nucleotide polymorphism (SNP) markers to study a wide geographic area along latitude (40–60 degrees) and elevation (350–3000 m) in British Columbia and Southwest United States. Our objectives were to characterize the genetic architecture and allele frequency differentials along latitude and elevation using neutral SSR markers and to depict fine-scale patterns of interspecific gene flow by studying the ancestry and interspecific heterozygosity of hybrids and by identifying the main types of selection that deviate SNP genomic clines from neutral expectations.

## Materials and methods

### Sample collection

**Figure 1:**
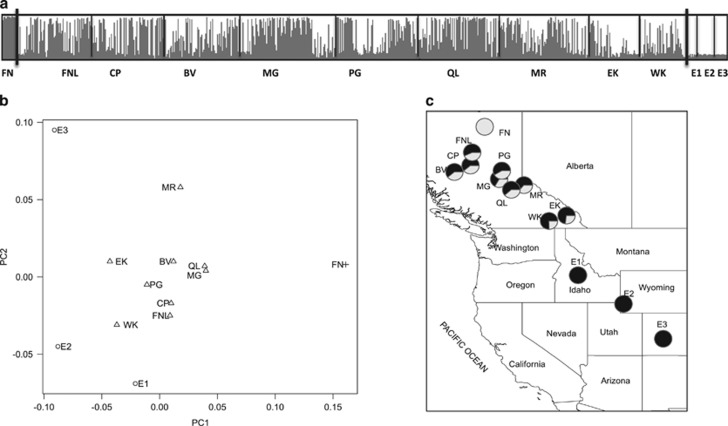
Analysis of population structure in the *P. glauca* × *P. engelmanni* hybrid zone. (**a**) Posterior estimates of cluster membership for *K*=2 in *TESS* based on 10 SSR markers. Populations are ordered by decreasing latitude from left to right. Population names corresponding to two- or three-letter codes can be found in Table 1. (**b**) Plot of first two principal coordinates (PC1 and PC2) based on Nei's genetic distances among populations. Circles represent *P. engelmannii* populations; triangles, hybrid populations; and a cross, a pure *P. glauca* population. (**c**) Geographical distribution and degree of admixture for populations of *P. glauca*, *P. engelmannii* and their hybrids. Black circles represent *P. engelmannii* and gray circles, *P. glauca* populations. Bi-color circles represent hybrid populations.

Fresh needle tissue from 805 individuals from 13 populations (nine allopatric and four sympatric) of white spruce, Engelmann spruce and their hybrids were sampled for DNA extraction (Figure 1, Table 1). In order to use previously collected, available genetic resources, needles were collected from grafted individuals cloned from trees in natural populations and archived in clone banks established by the British Columbia Ministry of Forests, Lands and Natural Resource Operations. In British Columbia, seed is managed for reforestation within geographic areas called seed planning zones (SPZ), and within each SPZ, seed transfer in this ‘interior spruce' complex is controlled by elevation rather than by species composition. In this study, we define a population as comprising those individuals that occur within the same SPZ. A subset of these populations (PG, QL, EK, WK and MR) was SNP-genotyped as previously described in De La Torre *et al.*, 2014b.

### DNA extraction and genotyping

Needle tissue was stored at −80 °C before isolation using the CTAB protocol (Doyle and Doyle, 1987). DNA quality and concentration were assessed visually using 0.8% agarose gels and quantified based on Nanodrop 2000C Spectrophotometer readings (Thermo Fisher Scientific Inc., Waltham, MA, USA). Polymerase chain reactions (PCRs) were performed using a PTC-100 thermal cycler (MJ Research Inc., Waltham, MA, USA). Each reaction had a total volume of 10 μl and contained 20 ng of nuclear DNA, 1 μl of 2 mM dNTP, 1 μl 10 × Paq5000 Reaction Buffer, 1 U Paq5000 DNA Polymerase, 0.5 pmol of M13 Infrared Label Primer and 1 pmol each of forward and reverse tailed primers. Samples were amplified using a modified version of Rungis' (2004) protocol, in which the annealing temperature varied according to each primer (Supplementary Table S1 in Supplementary Information). The PCR profile involved 2 min at 95 °C initial denaturation step, followed by 30 cycles of 95 °C for 20 s, appropriate annealing temperature for 20 s, 72 °C for 30 s and an extension cycle of 3 min at 72 °C. Following amplification, 3 μl of loading dye was added to each reaction. Amplification products were electrophoresed on a LI-COR 4200-automated sequencer using 7% polyacrylamide gels (Long Ranger TM, BioWhittaker Molecular Applications, Rockland, ME, USA). Amplification bands were scored using the Saga 3.3 automated microsatellite analysis software (LI-COR Inc., Lincoln NE, USA).

Twenty-five published nuclear microsatellite SSR loci (Rungis *et al.*, 2004) were tested for resolution and level of polymorphisms in 0.8% agarose gels. These markers have been widely used as neutral markers in a number of studies using the same or different plant species (Hamilton and Aitken, 2013; Baskauf *et al.*, 2014). Of the 25 microsatellites tested, 17 were successfully amplified, but only 15 exhibited polymorphisms. Of these, five primers were eliminated because they produced amplifications with multiple stutter bands, making scoring difficult. A total of 10 microsatellites were chosen that were polymorphic, amplified and scored reliably (Supplementary Table S1 in Supplementary Information). To test for the presence of genotyping errors and null alleles in the data set, the program MICRO-CHECKER (Van Oosterhout *et al.*, 2004) was used. Null alleles and possible stuttering were detected in SSR09 and SSR10, and null alleles were found in SSR03. These loci were re-scored before genetic analyses.

A subset of 750 individuals were SNP-genotyped at the Genome Quebec/McGill Innovation Centre using an Illumina bead array chip (Illumina Inc., San Diego, CA, USA) in conjunction with the GoldenGate allele-specific assay in a 96-well, 768 SNP format. White and Engelmann spruce samples were tested using 1536 SNPs from a large panel of candidate genes putatively involved in cold hardiness (Holliday *et al.*, 2008); insect herbivory resistance; and growth and bud set timing. A total of 86 SNPs were selected for this study. Criteria for SNP selection are explained in detail in De La Torre et al, 2014b.

### Genetic architecture based on SSR markers

Admixture proportion (*Q*) was estimated from nuclear SSR data using the Bayesian clustering approach implemented in STRUCTURE version 2.2 (Pritchard *et al.*, 2000). Models with a putative number of clusters (*K*) from 1 to 10 were tested using 50 000 iterations for the pre- and post-burn periods using the admixture model. Using a larger number of post-burn iterations (100 000) did not change the results obtained. The degree of admixture, *α*, was inferred from the data. Each run was repeated 20 times in order to estimate *K* using the method developed by Evanno *et al.*, (2005) with the program STRUCTURE HARVESTER version 0.6.7 (Earl and VonHoldt, 2011). Individuals with admixture proportions *Q*>0.9 from STRUCTURE results for either species were used as reference for ‘pure species' in hybrid index calculations using the INTROGRESS 1.1 (Gompert and Buerkle, 2010) package in R 3.0.3 (R Core Team, 2014).

In order to confirm the suitability of our selected *Q*-value threshold, and also to test the efficiency and accuracy of the STRUCTURE results, we used the results of an earlier genomic analysis based on 384 SNPs for the same hybrid zone (De La Torre *et al.*, 2014b). Assignment of pure parental species and hybrids was performed using STRUCTURE and NewHybrids (Anderson and Thompson, 2002) programs. Interestingly, both STRUCTURE and NewHybrids showed similar results, supporting the cutoff criteria used (De La Torre *et al.*, 2014a). To overcome possible biases that might have resulted from the inclusion of only one white spruce parental population, we included an additional white spruce population from Eastern Canada (De La Torre *et al.*, 2014a). The results of STRUCTURE (levels of admixture and asymmetric introgression) were consistent with the results using only one white spruce population. At the end, we decided not to include the Eastern Canada population because of its geographic remoteness to the studied area.

Population genetic structure was also assessed using the program TESS version 2.3 (Chen *et al.*, 2007). The admixture model was performed for values of *K*_max_ ranging from 2 to 14. Markov chain Monte Carlo algorithms were run for a length of 50 000 sweeps with burn-in periods of 30 000 sweeps. Each run was replicated 30 times. The 20% lowest deviance information criterion runs were averaged and plotted against the number of clusters (*K*_max_) to evaluate which *K*_max_ provided the best fit with the genetic data. On the basis of the results of the Evanno test, alignments of clusters for *K*=2 for STRUCTURE and TESS were optimized using the program CLUMPP 1.1.2b (Jakobsson and Rosenberg, 2007) and graphed with the program DISTRUCT (Rosenberg, 2004).

Global and pairwise population F_st_ values were calculated using GENALEX (Peakall and Smouse, 2006). Differences between and within populations and between species were estimated with analysis of molecular variance using GENALEX. Pairwise population F_st_ and Nei's genetic distances were used as input for a principal coordinates analysis in which the distance matrix was converted into a covariance matrix and data were standardized (divided by the square root of *n*−1) in GENALEX. Principal coordinates 1 and 2 were plotted using R 3.0.3 (R Core Team, 2014). To test for isolation by distance, Pearson's product–moment correlations were estimated between populations using genetic and geographical distances in Mantel tests based on 1000 permutations, using the Vegan package in R 3.0.3 (R Core Team, 2014). Genetic distance was expressed as F_st_/(1−F_st_).

### Geographic and genomic cline analysis

Hybrid zones are often well described by a sigmoid curve showing the expected allele frequencies along geographic axes. Cline analysis can be used to estimate the shape, location (center) and width of the cline, and to test for concordance and coincidence in these parameters between markers (Gay *et al.*, 2008). In this study, the best-fitting curve was estimated for each of the 10 microsatellites and 86 SNP markers using a maximum likelihood approach implemented in the program CFit-6 (Gay *et al.*, 2008). To reduce the observed microsatellite allelic variation to two allele systems, each allele was assigned to a species-specific compound allele according to their coordinates in a multiple correspondence analysis in GENETIX (Belkhir *et al.*, 1998). Runs were repeated several times using different seeds to ensure accurate results. The position of the cline center, defined as the point of the steepest slope, and the width, defined as the inverse of the maximum slope, were estimated for each marker. To determine whether cline location varied significantly among the markers, we used Akaike information criterion values to compare the likelihood of a model in which all clines vary independently and a model in which all clines have the same center. The geographic distance was calculated from north to south, beginning with the northernmost population sampled (Fort Nelson), following the specifications of the CFit-6 program. Geographical clines in which SNP allele frequencies varied across elevational gradients were also evaluated.

Genomic clines were calculated from SNP markers using the parametric procedure with 1000 permutations with the *INTROGRESS* 1.1 (Gompert and Buerkle, 2010) package in R 3.0.3 (R Core Team, 2014). This method uses multinomial regression to predict the probability of a given genotype (homozygous Engelmann (EE); heterozygous (WE) or homozygous white (WW)) for a marker as a function of hybrid index between a pair of species (Gompert and Buerkle, 2010). Expected genomic clines based on a null model are used as a reference to identify loci that deviated from neutral expectations (*P*<0.0001). Evidence for directional selection for homozygous genotypes corresponds to an increase (+) or decrease (−) in the observed probability density in comparison with neutral expectations, resulting in genotypes EE−, EE+, WW+ or WW−. In the same way, an increase (+) or decrease (−) in the observed probability density of heterozygous genotypes will be observed as ‘overdominance' (WE+), or ‘underdominance' (WE−), respectively. A combination of increased density of one homozygous genotype, whereas the other homozygous and heterozygous genotypes remain neutral may be indicative of epistasis (Gompert and Buerkle, 2009). Congruence between geographic and genomic clines was used to identify loci that may be involved in reproductive barriers between species. When congruence was observed, loci with generally narrow geographic clines and genomic distributions away from neutral expectations were identified. These selected loci were tested for associations between allele frequencies and previously identified environmental variables such as mean annual temperature, precipitation as snow (PAS), mean annual precipitation (MAP) and summer heat moisture index (De La Torre *et al.*, 2014a). Initial attempts to perform genomic cline analyses based on SSR data showed unreliable results because of a very large variation in the hybrid index and interspecific heterozygosity. We interpret this limitation as the lack of power of SSR because of the small number of markers to differentiate between advanced generation hybrid classes in the hybrid zone. For this reason, only genomic clines based on SNP data were analyzed in this study.

### Simulations: ancestry and interspecific heterozygosity

To illustrate how the joint distribution of ancestry and interspecific heterozygosity change in generations following initial admixture of parental populations, we used the software *quantiNEMO* (Neuenschwander *et al.*, 2008) to simulate data under simplified assumptions of non-overlapping generations and random mating between hermaphroditic adults within each resulting patch. Each simulation consisted of nine populations that were arrayed in a linear manner and with stepping-stone migration, that is, migration strictly occurred between neighboring populations. The sole exception was a set of simulation runs aimed assessing the impact of island model of migration, where migration was occurring at the same rate as in the stepping stone simulations, but where migrant individuals could enter any patch regardless of spatial location. In all simulations, local carrying capacity of each population was set to 1000 individuals.

For all simulations we modeled a single quantitative trait where the trait optimum varied in a clinal manner across the set of populations, the optimum mean phenotype in the different populations were set to −5, −5, −4, −2, 0, 2, 4, 5, 5. The quantitative trait was assumed to be controlled by 40 bi-allelic loci, where the alleles increased or decreased the phenotypic value of the trait by one unit (allelic effects of −1 and 1, respectively). The trait was assumed to have a constant heritability of 0.5 and fitness W for an individual with phenotype P in each local population; and it was modeled as stabilizing selection around the local optima as *Z*_opt_:


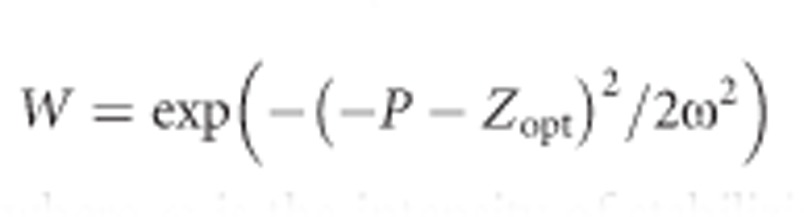


where *ω* is the intensity of stabilizing selection. All simulations used *ω*=10. In addition to the quantitative traits, we also simulated data for 40 bi-allelic neutral loci. Each simulation was initiated with three populations fixed for alleles representative of each parental species (populations 1, 2 and 3 and 7, 8 and 9, respectively) and the three remaining populations were colonized through migration from neighboring populations. After simulations had finished, the data were read into R and analyzed using the package INTROGRESS 1.1 (Gompert and Buerkle, 2010).

### Patterns of allelic variation across latitude and elevation

GENALEX version 6.4 (Peakall and Smouse, 2006) was used to calculate allele frequencies, patterns of variation across populations (number of effective, local and private alleles) and genetic diversity (observed and expected heterozygosity) along elevational and latitudinal gradients. Allelic richness was computed using rarefaction, which accounts for the heterogeneity in sample sizes, with the program FSTAT (Goudet, 2001). *χ*^2^ goodness-of-fit tests were performed to measure deviations from Hardy–Weinberg equilibrium. Inbreeding coefficients for each population and each locus were estimated with the program GENEPOP version 4 (Rousset, 2008). Exact *P*-values were calculated using the Markov chain method with 10 000 iterations.

## Results

### Genetic architecture and introgression based on SSR markers

Bar plots of posterior estimates of cluster memberships and the frequency distribution of hybrid classes revealed an extensive hybrid zone with a wide variety of hybrid classes, in which hybrid composition exhibited clines along latitude and elevation corresponding to climatic gradients in temperature and precipitation (Figure 1; Supplementary Figure S1). Within the latitudinal range sampled, SNP hybrid index estimates suggest that Engelmann spruce mainly occurs above 1800 m and white spruce below 600 m (Supplementary Figure S1).

Hybrid individuals collectively showed asymmetry in introgression (evidenced by a shift of the peak of the histogram towards Engelmann spruce, Supplementary Figure S2), meaning that in general hybrids had a greater genetic contribution from Engelmann spruce than from white spruce. In most hybrid populations, there were more individuals in the Engelmann-backcross classes (hybrid index 0.8–0.9) than in the white-backcross classes (hybrid index from 0.1 to 0.2; Supplementary Figure S2). Hybrid populations varied in their levels of admixture and genetic distances from pure species according to their latitude. Hybrid populations in mountainous southern British Columbia (East Kootenay and West Kootenay), where elevations are higher, on average, had substantially higher ancestry from Engelmann spruce. Populations in less mountainous, lower elevation areas in central British Columbia (Finlay and Prince George) had more equal ancestry from both species.

On the basis of SSR data, we found that TESS, STRUCTURE and F_st_ estimates, showed clear differentiation between white and Engelmann spruce. Mantel tests revealed strong associations between geographical and genetic distances (*r*=0.5712, *P*<0.001), and between elevation and genetic distance (*r*=0.5446, *P*<0.006; Supplementary Figure S3). The genetic structure of populations was also evidenced by results of the PCA (Figure 1). Principal coordinate (PC) 1, which explained 52.4% of the variation in the data, clearly separated Engelmann and white spruce. PC1 also separated the hybrid populations East Kootenay and West Kootenay, which are genetically closer to Engelmann spruce, from other hybrid populations. Most of the hybrid populations (with the exception of West Kootenay, East Kootenay and Mount Robson) clustered together at the union of PC1 and PC2. PC2, which explained 22.6% of the variation in the data, differentiated Engelmann populations E1 (Salmon River, Idaho) and E2 (Teton Wasatch, Wyoming) from E3 (Fishlake Lasal, Colorado). All hybrid populations were clearly differentiated from the pure parental populations. Salmon River and Teton Wasatch (Engelmann) populations were genetically more similar to hybrid populations than Fishlake Lasal (Engelmann spruce) and Fort Nelson (white spruce).

### Variation in ancestry and interspecific heterozygosity in admixed populations

**Figure 2:**
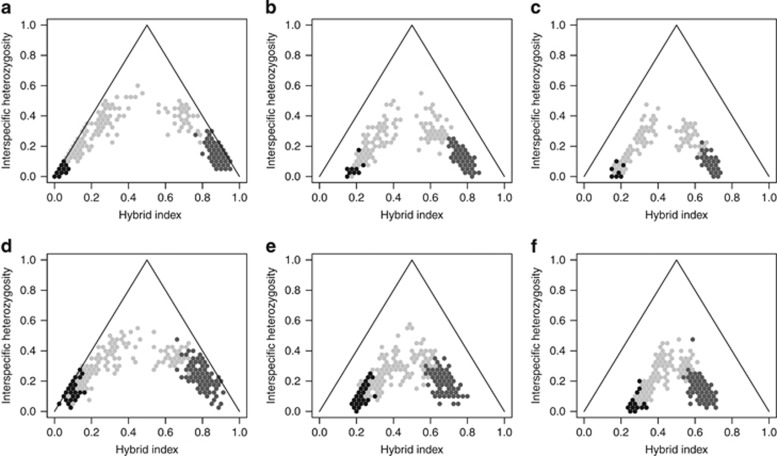
Effect of different proportions of neutral/selected loci on the ancestry and interspecific heterozygosity of parental and hybrid individuals. Simulations were carried out using (**a**, **d**) 40 neutral/0 selected (**b**, **e**) 30 neutral/10 selected (**c**, **f**) 20 neutral/20 selected, **a**–**c**: 100 generations, **d–f**: 3000 generations. Parental species are represented by black and dark gray dots, hybrids, in gray.

To illustrate how the joint distribution of ancestry and interspecific heterozygosity changes in the generations following initial admixture of parental populations, we ran simulations under different scenarios. In the first scenario, we were interested in knowing whether our measurements of ancestry and interspecific heterozygosity derived from candidate gene SNPs differed from distributions under neutrality. Interestingly, we found that average patterns of ancestry and interspecific heterozygosity change very little when the number of selected loci is lower than 30% (Figure 2). When comparing simulations between 40 neutral/0 selected and 36 neutral/4 selected, only one parental species showed significant differences in ancestry (*χ*^2^=45.9, *P*-value=3.367*e*^−06^); however, the other parental species and hybrids showed no significant differences (*P*-value>0.001). Neither parentals nor hybrids showed differences in interspecific heterozygosity (*P*-value>0.001). When loci under selection represent 30–50% of the loci under study, the interspecific heterozygosity decreases (*P*-value<3.994*e*^−11^) and the hybrid index (*P*-value<2.2e^−16^) decreases in range. After 3000 generations following initial admixture, with 50% of loci under selection, the differentiation between parentals and hybrids is lost, and the hybrid zone becomes a hybrid swarm (Figure 2).

**Figure 3:**
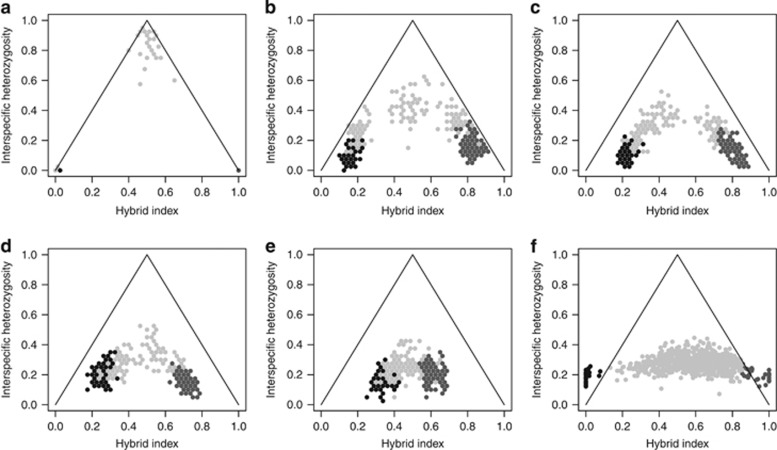
Relationship between ancestry and interspecific heterozygosity in parental and hybrid individuals at different time periods, based on simulated and experimental SNP data. Simulations were carried out for neutral loci at (**a**) 10 generations, (**b**) 100 generations, (**c**) 1000 generation, (**d**) 3000 generations and (**e**) 10 000 generations. Plot F shows experimental data from 86 SNPs and 711 individuals of *P. glauca* (black dots), *P. glauca* × *P*. engelmannii hybrids (gray dots) and *P. engelmannii* (dark gray dots).

In a second scenario, we simulated variation in ancestry and interspecific heterozygosity over time (Figure 3). In the early stages of hybridization (generation 10), hybrids will have a maximum interspecific heterozygosity of 1, and ancestry value of 0.5, as a result of equal allelic contributions between parental species. In subsequent generations, there will be less differentiation between parental and hybrids as a consequence of increased interspecific gene flow. Interspecific heterozygosity of hybrids as well as the ancestry levels will tend to decrease. It will take many generations (10 000) to reach a low level of interspecific heterozygosity of 0.4. We found a similar distribution of ancestry of hybrids in simulated populations under 100 (*χ*^2^=834, *P*-value=0.037) and 1000 (*χ*^2^=834, *P*-value=0.002) generations to those of our SNP data.

In the third scenario, we ran simulations to assess the impact of the island model of migration on the levels of ancestry and interspecific heterozygosity. Migration was set to occur at the same rate as in the stepping stone simulations; however, migrant individuals could enter any patch regardless of spatial location in all simulations. When migration occurred only between adjacent populations (stepping stone model), the patterns of ancestry and interspecific heterozygosity varied little even after 3000 generations following initial contact. On the other hand, with migration under the island model, the hybrid zone tends to merge into a big hybrid swarm as early as 100 generations (Supplementary Figure S4). When comparing the simulation results with our experimental SNP data, we conclude that gene flow is likely similar to stepping stone migration in the white spruce × Engelmann spruce hybrid zone. The strong pattern of isolation by distance observed across this hybrid zone further support this conclusion.

### Geographical and genomic cline analysis

Our results indicate the presence of an extensive hybrid zone between white and Engelmann spruce between 50 and 56°N and 600–1800 m in elevation in Western Canada and the United States. Both SSR and SNP clinal analyses revealed a complex hybrid zone, in which different loci introgress in different directions, with the center of individual clines shifted either toward Engelmann or white spruce. SSR clinal analysis pointed toward a unimodal hybrid zone distribution, whereas the SNP clinal analysis showed differentiation between parental and hybrids' habitats, pointing toward a trimodal hybrid zone distribution (Supplementary Figure S1).

SSR cline width varied from 50.1 to 401 km, with seven of the ten loci having cline widths between 50 and 59.82 km, and the remaining three loci with variable widths over 100 km (Table 2). The cline center varied between 0.01 and 1999 (km south of the northern population (Fort Nelson), with a mean of 509.61 km across all loci. SNP cline width varied between 50 and 2295 km, with seven loci having narrow widths between 50 and 50.44 km; 30 loci having widths between 51 and 100 km; and 18 loci having wider widths over 100 km. Cline center varied from 0 to 1999 km with a mean of 483.12 km. Both SSR and SNP results pointed toward a mean cline center located between Prince George and Mount Robson (53.16–53.94°N, 119.93–121.95°W). In 18 of the 86 SNP loci, neither geographical nor genomic cline parameters could be estimated because of low genetic differentiation between parental species. These loci were not considered for subsequent analyses.

**Figure 4:**
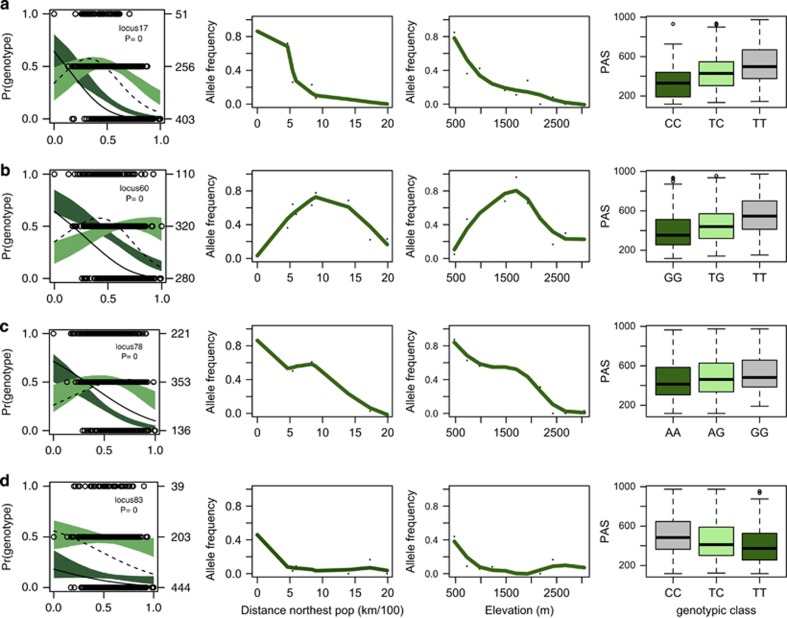
Genomic, geographic and climatic clinal analysis of the loci that deviated from neutrality in the *P. glauca* × *P. engelmannii* hybrid zone and suggested modes of selection, based on 86 SNP. *Left graphs*: solid colored regions represent the 95% confidence intervals for A_1_A_1_ (dark green) and A_1_A_2_ (light green) genomic clines, given neutral introgression. The solid and dashed lines denote fitted genomic clines from observed genotype frequencies for A_1_A_1_ and A_1_A_2_ genotypes, respectively. Empty circles indicate the raw genotype data, with counts of individuals on the right vertical axis. Hybrid index indicates the fraction of alleles derived from *P. engelmannii* population, where ‘0' is pure *P. glauca* and ‘1' is pure *P. engelmannii*. *Middle graphs*: geographic clines describe changes in allele frequencies along latitude (distance from the northernmost population, Fort Nelson) and along elevation*. Right graphs*: boxplots describe associations between genotypic classes and PAS in each of the SNPs studied. All associations are significant (*P*<0.001). Dark green boxplots describe white spruce genotypes; light green, hybrid genotypes; and gray, Engelmann genotypes. SNP gene annotations and modes of selection are as follows: (**a**) SNP 13_496 (FK506-binding protein) shows deviation from neutrality because of directional selection toward Engelmann spruce; (**b**) SNP 288_302 (late elongated hypocotyl) is an example of overdominance; (**c**) SNP 85_279 (annexin) shows directional selection toward white spruce; (**d**) SS_CO483349-contig3-358 shows directional selection toward Engelmann spruce.

The genomic cline analysis identified 45 SNP loci that deviate from neutral expectations (*P*>0.0001). Overdominance (heterozygous genotypes favored over parental genotypes) and directional selection (one parental genotype is favored over the other parental and hybrids) were the main types of selection explaining deviation of genomic clines away from neutral expectations (Supplementary Table S5). A total of 18 loci showed patterns of overdominance, 13 showed patterns of directional selection toward white spruce and 8 of directional selection toward Engelmann spruce. We also observed five loci deviating from neutrality as a result of underdominance (selection against heterozygous individuals), and one case of epistasis. Because the parametric procedure used for genomic cline estimation can lead to an increase in the rate of false positives when spatial populations structure is present in the hybrid zone, we compared the results of the genomic clines with the geographic clines. Within SNP clines with narrow widths (cutoff of <51 km was based on SSR narrowest clines), directional selection toward either Engelmann or white spruce was observed in three of the seven loci (85_279_S, 13_496_NS, SS_CO483349-contig3-358); and overdominance was observed in locus 288_302_NS (Table 3). All of these loci showed significant associations with the MAP, and PAS; and some loci showed significant associations with summer heat moisture index and mean annual temperature (Figure 4; Supplementary Figure S5 and Table S6).

### Patterns of allelic variation across latitude and elevation

**Figure 5:**
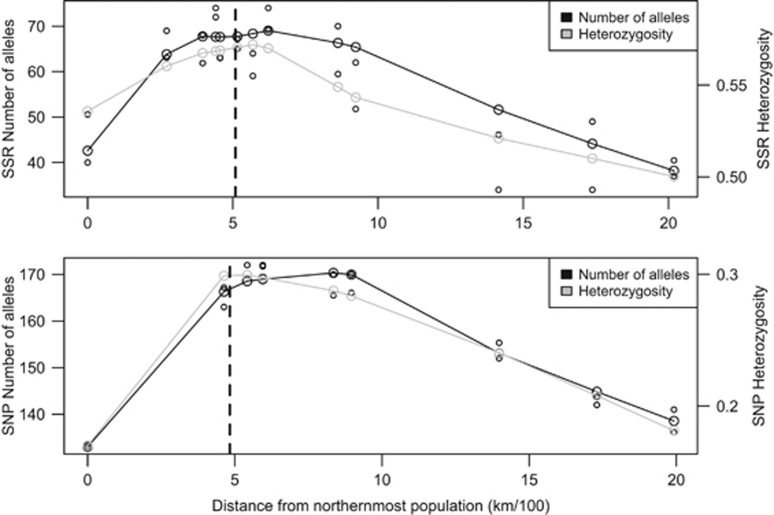
Genetic estimates along latitudinal distance in the *P. glauca* × *P. engelmannii* hybrid zone based on SSR and SNP markers. Number of different alleles (*N*_a_) and heterozygosity (*H*_e_) were plotted against distance (kg100^–1^) from the northernmost population sampled (Fort Nelson). Dotted black lines indicate the mean center of the hybrid zone for each SSR and SNP markers.

Whereas all populations were highly diverse for the microsatellite and SNP markers assayed, those from the introgression zone at intermediate elevations and latitudes had, on average, more alleles and tended to have higher levels of heterozygosity and allelic richness (Figure 5; Supplementary Tables S3 and S4 in Supplementary Information). The number of alleles (*N*_a_) followed a bell-shaped distribution, with the more central Quesnel population having the highest SSR and SNP diversity. The peak in *N*_a_ is shifted slightly toward Engelmann spruce (south from the average center of the hybrid zone) in both SSR and SNP clinal analyses (Figure 5). The number of alleles at intermediate elevations (850–1350 m) was twice as high as the number of alleles found at elevations corresponding to pure species (40 alleles at 350–600 m and 41 alleles at >1800 m). Considering that the number of alleles with a frequency higher than 5% was not very different among populations, the *N*_a_ mainly reflects the presence of low-frequency alleles (both rare and private) in the hybrid populations. Somewhat surprisingly, private alleles were only found in hybrid populations originating between 600 and 1600 m of elevation, and not in parental populations.

Heterozygosity was highest in the McGregor population (*H*_e_=0.592) when analyzing SSR diversity, and in nearby Mount Robson (*H*_e_=0.307) when analyzing SNP diversity. Showing a similar pattern to *N*_a_, the peak in heterozygosity was also shifted toward Engelmann spruce (south of average center of the hybrid zone) for SNP markers compared with SSRs (Figure 5). The analysis of molecular variance found that most of the variation (93%) occurred within populations with only 3% of the variation among populations and 4% between species. Correspondingly, F_st_ values between white and Engelmann spruce were relatively low, with the average of all pairwise F_st_ of 0.092±0.003 s.d. for SSR markers, and 0.188±0.02 s.d. for SNP markers (Supplementary Table S2). Despite their relatively low values, pairwise interspecific F_st_ estimates were higher between allopatric than between sympatric pure parental species populations (*t*=6.1385, df=5.51, *P*-value=0.001174).

## Discussion

### Genetic architecture of a wide and complex hybrid zone

The genetic architecture of natural hybrid zones provides a unique opportunity to study the interplay between gene flow as homogenizing force and natural selection that promotes population divergence (Morjan and Rieseberg 2004). The genetic structure of the white × Engelmann spruce hybrid zone revealed an extensive genetic complex shaped by introgressive hybridization and environmental gradients. Extensive introgression was observed, where the majority of individuals in the zone sampled have hybrid ancestry and most alleles are shared by both pure species and hybrids (Figure 1). Given the recent divergence between these two closely related species (Bousquet *et al.*, 2007), allele sharing could have resulted from the retention of ancestral polymorphisms and incomplete lineage sorting. However, ancestral polymorphisms alone could not produce the patterns of gradual changes in genotype frequencies observed across latitude or elevation. Given the extension of introgression, and that sympatric populations showed less genetic divergence than allopatric populations, interspecific gene flow seems to be a more plausible explanation. The spatial patterns of allelic richness and heterozygosity are also indicative of introgression as a result of secondary contact.

In most hybrid zones, clines are characterized by their narrow width, consistent shape and close concordance, suggesting that clines are maintained by dispersal and selection against heterozygotes (tension zones). In our study, however, the lack of concordance between markers, the width of the clines and the abundance of advanced-generation hybrids suggest that the Engelmann × white spruce hybrid zone is not a tension zone, and supports our recent studies suggesting the Engelmann × white spruce hybrid zone is maintained by a bounded hybrid superiority model (De La Torre *et al.*, 2014a). Our study indicates that, despite high levels of introgression, white and Engelmann spruce have maintained their species identities (evidenced by the F_st_, STRUCTURE and principal coordinates analysis results).

### Ancestry versus interspecific heterozygosity

Our study has revealed the permeability of spruce genomes to introgression in this hybrid zone, with a broad range of ancestry and low interspecific heterozygosity. When comparing our SNP data with the results of the simulations, we find similar levels of ancestry and interspecific heterozygosity in simulated populations between 100 and 1000 generations (Figure 3). Considering nonoverlapping generations of 50 years each, this would mean that the hybrid zone is between 5000 and 50 000 years old. This is coincident with our recent paleoclimatic modeling that suggests that white spruce and Engelmann spruce have a long history of introgression, which likely dates to at least 21 000 years before present (De La Torre *et al.*, 2014b).

The hybrid zone follows stepped elevational and latitudinal clines in admixture. Stepped clines, in which allele frequencies vary considerably over short geographic distances, are caused by differential selection and population substructure (Barton and Hewitt, 1985). In the white spruce × Engelmann spruce hybrid zone, isolation by distance implied that geographic distances along elevation and latitude might act as barriers to gene flow. The results of the simulations also suggest that interspecific gene flow is mainly occurring between adjacent populations along latitude and elevation (stepping stone model) rather than indiscreetly among patches (Supplementary Figure S4). This suggests that genetic drift may be limiting interspecific gene flow. However, the high density of individuals in the studied zone and the presence of long-distance gene flow between species make drift alone unlikely to explain the pattern observed. The association of narrow geographic clines with certain environmental variables, and the presence of locally adapted hybrids and parentals to different environmental niches, points toward a hybrid zone shaped by environmental selection (De La Torre *et al.*, 2014a). A combination of both isolation by distance and isolation by environment is probably the most likely scenario.

### Overdominance and directional selection deviate clines from neutrality

A close examination of genetic clines can improve our understanding of selection and gene flow across hybrid zones (Jiggins and Mallet 2000). The frequency distribution of hybrid classes and the admixture clines based on 86 SNP markers indicate the presence of a trimodal hybrid zone (Jiggins and Mallet, 2000; Gay *et al.*, 2008), in which hybrids form a clearly identifiable group characterized by intermediate allele frequencies along elevational and latitudinal gradients, broad genetic variance due to varying levels of introgression and weak selection against hybrids (Supplementary Figure S1). This distribution supports recent studies that indicate that white, Engelmann and hybrids are adapted to different habitats as a consequence of strong environmental selection acting along elevation and latitude (De La Torre *et al.*, 2014a), but differs from previous studies suggesting extensive admixture and the absence of genetic differentiation between parental species (Rajora and Dancik 2000). The use of a small numbers of markers may lead to assignment errors in advanced generation hybrids, leading to failure to depict fine-scale admixture patterns. Given the complexity of the hybrid zone, in which advanced-generation hybrids predominate, at least 48 markers are needed to differentiate backcrosses from pure species (Vähä and Primmer, 2006). The difference between the distribution of admixture based on SSR (unimodal) and SNP (trimodal) markers may be due to the lack of power of SSR results as a consequence of the small number of markers.

The outcomes of admixture between two distinct species can vary from gene to gene. Different alleles may be favored in different environments or genetic backgrounds and maintained by selection in the presence of random mixing (Barton and Gale, 1993; Hancock *et al.*, 2011). These alleles are expected to introgress less than neutral markers, resulting in narrow cline widths, and contributing to reproductive isolation between species (Teeter *et al.*, 2008). In our study, we identified seven SNP loci with narrow cline widths (width <51 km; Table 3). Two of these loci (13_496_NS and 68_286_S) were previously identified as strong candidates for adaptation in a 311-SNP study using F_st_ outlier methods (De La Torre *et al.*, 2014b). Congruency was observed between geographical and genomic clines in four (13_496_NS, 288_302_NS, 85_279_S, SS_CO483349-contig3-358) of the seven loci with narrow widths. In these loci, overdominance and directional selection were found to be the main factors responsible for shifting genomic clines away from expectations under neutrality (Figure 4).

Overdominance has been suggested as an explanation for increased fitness in hybrids (Rieseberg *et al.*, 1999). Caused by the non-additivity of allelic effects within a locus, overdominance seems to be more important in first-generation hybrids (heterosis) than in advanced-generation crosses (transgressive segregation; Rieseberg *et al.*, 1999). Increased hybrid fitness as a result of co-adapted gene complexes has also been suggested in a number of recent studies (Pritchard *et al.*, 2011; Lindtke *et al.*, 2012). In this study, evidence of overdominance was found for locus 288_302_NS, suggesting that this locus may contribute to the increased hybrid fitness previously reported in the hybrid zone (De La Torre *et al.*, 2014a). Functional annotation of SNP 288_302_NS points toward a homolog of the *Late Elongated Hypocotyl* (*LHY*) gene, part of the molecular circadian clock that provides the timing mechanism required for the response to seasonal changes in photoperiod. In *Arabidopsis*, mutation of this gene caused disruption of the circadian clock regulation of gene expression and caused flowering to occur independently of photoperiod (Schaffer *et al.,* 1998). In *Populus*, LHY is an important locus explaining natural variation in dormancy and bud phenology (Ibanez *et al.*, 2010).

Directional selection toward white spruce was observed for locus 85_279_S, whereas selection toward Engelmann spruce was detected in loci 13_496_NS, SS_CO483349-contig3-358. A signature of directional selection may be an indication of adaptive introgression from one parental species to the hybrids. Adaptive introgression resulting in excess ancestry in hybrids coupled with increased genetic differences between parental species can help identify important loci involved in local adaptation (Gompert and Buerkle, 2009; Nolte and Tautz, 2010; Gompert *et al.*, 2012), and is likely an important process in promoting adaptive differences between white spruce, Engelmann spruce and their hybrids (De La Torre *et al.*, 2014a). Functional annotation of SNP 13_496_NS points toward an *FK506*-binding protein, which is encoded by a small gene family in higher plants and which may have an important role in plant signal transduction (Luan *et al.*, 1994). Annexins (SNP 85_279_S) have an important role in calcium signaling, an important process in transcriptional and post-transcriptional regulation of cold-stress gene expression (Qin *et al.*, 2011). The same gene was reported in a previous study of cold adaptation in *P. sitchensis* in British Columbia (Holliday *et al.*, 2008).

Our results indicate that all four SNPs with narrow clines and deviations from neutrality were strongly associated with local estimates of PAS and MAP (Figure 4; Supplementary Figure S5 and Table S6). Higher values of PAS (PAS>500 mm) and MAP (MAP>1000 mm) were associated with high-elevation, cold and humid Engelmann habitats, whereas lower values of PAS (<400 mm) and MAP (<900 mm) were associated with lower-elevation, drier and warmer in summer white spruce habitats. The intermediate values of PAS and MAP were associated with hybrid habitats. These genes may contribute to increased fitness of Engelmann spruce (SNP 13_496_NS, FK506-binding protein), white spruce (SNP 85_279_S, annexin) and hybrids (SNP 288_302_NS, late elongated hypocotyl) in their home environments, as it is expected that after prolonged hybridization only selected genes (or those that interact epistatically with directly selected genes) conferring an adaptive advantage in one environment would be associated with that environment (Barton and Gale, 1993). Our results are coincident with our recent findings suggesting that the white × Engelmann spruce hybrid zone is maintained by adaptation to the length of growing seasons and the persistence of the snowpack, following a bounded hybrid superiority model (De La Torre *et al.*, 2014a).

At other loci, advantageous alleles have spread faster and thus further than neutral alleles from one species to another, resulting in wide cline widths. These loci with wide clines (>1000 km) were associated with various important functions including carbohydrate and lipid metabolism, calcium signaling and transcription regulation (Supplementary Table S5). Most of these clines showed deviations from neutrality because of directional selection toward either Engelmann or white spruce, reducing genetic differences between species.

### Asymmetric introgression may result from cline movement

Our results points toward asymmetry in introgression favoring Engelmann spruce ancestry, as previously postulated by other studies in the hybrid zone in Alberta (Rajora and Dancik, 2000), as well as in our analysis of 311 SNPs within candidate genes for local adaptation to climate (De La Torre *et al.*, 2014b). The asymmetry in introgression and the rarity of hybrid indices for individuals corresponding to hybrids backcrossed to white spruce is most easily explained by the apparent difference in population density between less abundant white spruce and more abundant Engelmann spruce. Hybrids likely have more opportunities to mate with Engelmann spruce than with pure white spruce just because Engelmann spruce is more abundant in the area. Population density has been associated with asymmetric introgression in other genera such as *Morus* (Burgess *et al.*, 2005). In addition, Engelmann spruce populations may use hybrid populations as stepping stones to move pollen down slope, in a similar way as *Quercus* populations disperse their pollen (Petit *et al.*, 2003). An alternative explanation is that offspring resulting from hybrids that are backcrossed to white spruce exhibit reduced hybrid fitness caused by the break-up of co-adapted gene complexes (Bateson–Dobzhansky–Muller genic incompatibilities). In fact, our results suggest the presence of underdominance in five SNP loci. The absence of hybrid individuals in parental species habitats also suggests some extrinsic postzygotic isolation such as that of the bounded hybrid superiority model of hybrid zone maintenance, in which hybrids are fitter than parentals in intermediate environments (De La Torre *et al.*, 2014a).

Another possible explanation for asymmetry in introgression is temporal isolation. Temporal isolation has been identified as the cause of asymmetrical introgression in several genera including *Populus* (Lexer *et al.*, 2005), *Iris* (Martin *et al.*, 2007) and *Quercus* (Bacilieri *et al.*, 1996). White spruce and Engelmann spruce are monoecious, having female and male strobili produced by individuals. Previous studies have indicated differences in vegetative primary growth phenology between white spruce and Engelmann spruce, with Engelmann spruce having earlier bud burst and bud set timing than white spruce in the same environment (De La Torre *et al.*, 2014a). In addition, some of the SNP loci associated with bud phenology showed outlier behavior, suggesting temporal isolation may be acting as a reproductive barrier between the species (De La Torre *et al.*, 2014b). Spring vegetative and reproductive phenologies are correlated in several spruce species (Major *et al.*, 2005). If vegetative bud break and reproductive phenology are correlated in this hybrid zone, when Engelmann spruce pollen is shed, white spruce female strobili in the same environment may not be receptive. However, female strobili of hybrids are likely receptive before white spruce, giving the hybrids a greater chance of being fertilized by Engelmann rather than by white spruce.

Although temporal isolation and differences in population density cannot be ruled out as causes of asymmetric introgression, the ancient nature of the hybrid zone suggests that the explanation for asymmetry may come from past changes in demography of the hybridizing species. The shift in allelic richness and heterozygosity toward Engelmann spruce suggests that the asymmetry in introgression has likely been caused by past cline movement (Figure 5). Pollen dispersal from Engelmann to white spruce would have moved the cline northward, leaving a peak of allelic richness behind where the previous cline center was first located. Repeated and more frequent backcrossing between the hybrids and that parental species produced advanced generation hybrids that resemble one of the parental species (Buggs, 2007). This would produce asymmetry in introgression as a consequence of hybrid zone movement. This hypothesis could explain why there are few white spruce backcrosses in the zone, and would suggest that the hybrid zone is moving towards white spruce. Our recent phylogeographic studies based on pollen records, climate data and SNPs from 311 candidate gene markers have found that this hybrid zone has likely moved in its early formation after the Last Glacial Maximum as a result of white and Engelmann's range expansion and contractions (De La Torre *et al.*, 2014b). The Sitka spruce (*P. sitchensis*) × white spruce hybrid zone is also asymmetric and appears to have moved from Sitka toward white spruce, in part due to the postglacial colonization sequence of the region (Hamilton and Aitken, 2013). Similar patterns of asymmetric introgression as a result of past demographic events due to Pleistocene climate changes was also found in other hybrid zones such as *Lepus granatensis* × *L. europaeus* (Melo-Ferreira *et al.*, 2007), and *Thomomys actuosus* × *T.b ruidosae* (Ruedi *et al.*, 1997). Cline movement as a consequence of more recent climate change was found in a chickadee hybrid zone (*Poecile carolinensis × P. atricapillus*; Taylor *et al.*, 2014).

The work described here is the last in a series of studies exploring the genetic and genomic basis of local adaptation to climate in the *P. glauca* × *P. engelmannii* hybrid zone in western North America. Our study has revealed the permeability of spruce genomes to introgression in this hybrid zone, in which different alleles are favored in different environments or genetic backgrounds and maintained by selection. By identifying populations with high-diversity levels and adaptations to particular environments, we set the ground for future studies aiming to predict the response of populations to climate change. This knowledge can then be translated into climate-based seed transfer recommendations to improve reforestation success, forest health and productivity for new climates.

## Data ARCHIVING

Microsatellite data are available at the Dryad Digital Repository: http://doi.org/10.5061/dryad.473n5. SNP data in available at the Dryad Digital Repository: http://doi.org/10.5061/dryad.7h65f.

## Figures and Tables

**Table 1:** Geographical coordinates and climatic variables for 805 individuals of *Picea glauca, P. engelmannii* and their hybrids

*Population*	*Province*	*Elevation (m)*	*Latitude (degree)*	*Longitude (degree)*	*MAT (degree)*	*MAP (mm)*	N
Fort Nelson (FN)*	B.C	300–580	58.4–59.4	120.5–126.3	0.29	509	22
Finlay (FNL)	B.C	677–1372	55–56.9	123.2–125.8	0.72	645	80
Central Plateau (CP)	B.C	671–1190	54.4–55.8	123–126	1.8	622	69
Bulkley Valley (BV)	B.C	647–1190	53.5–55.1	125.3–127.2	2.23	568	70
Prince George (PG)	B.C	610–1189	53.2–54.1	121.9–122.3	2.75	769.4	86
Mc Gregor (MG)	B.C	610–1372	53.9–55.3	120.9–122.9	2.3	1137	81
Mount Robson (MR)	B.C	701–1800	52.2–53.8	118.3–121.4	1.72	1166	50
Quesnel Lakes (QL)	B.C	670–1585	51.8–53.3	119.6–122.1	2.31	909	114
East Kootenay (EK)	B.C	1006–2012	49.2–50.8	115.1–116.6	1.89	962.7	86
West Kootenay(WK)	B.C	640–2225	49–50.4	115.3–119	2.45	1051.7	53
Salmon River (E1)**	Idaho	1859–2530	43.8–46.2	113.7–115.9	2.76	994.9	13
Teton-Wasatch (E2)**	Wyo.	1935–3292	40.4–43.8	109.5–111.6	2.51	843.4	35
Fishlake-Lasal (E3)**	Col.	2606–3383	37.5–39.8	109.2–112.8	3.51	735.7	46

Abbreviations: B.C, British Columbia; Col., Colorado; MAP, average mean annual precipitation; MAT, average mean annual temperature; *N*, sample size; Wyo., Wyoming.

Two- or three-letter codes are used to identify populations in subsequent tables and graphs. Reference populations are identified in the table (* for *P. glauca* and ** for *P. engelmannii*).

**Table 2:** Cline parameters and main genetic estimates for 13 *P. glauca*, *P. engelmannii* and hybrid populations based on 10 microsatellite loci

*Loci ID*	*Center (km)*	*Slope*	*Width (km)*	H_*t*_	*Mean* H_*e*_	*Mean* H_*o*_	*F*_*is*_
SSR01	1999	(−) 0.25	400.95	0.503	0.489	0.459	−0.018
SSR02	0.01	(−) 0.34	289.98	0.828	0.807	0.754	0.06
SSR03	0.01	(−) 1.74	57.35	0.718	0.688	0.667	0.007
SSR04	35	(+) 1.67	59.82	0.506	0.454	0.307	0.206
SSR05	0.02	(−) 1.97	50.74	0.74	0.69	0.73	−0.02
SSR06	441	(−) 1.81	55.27	0.628	0.582	0.445	0.095
SSR07	0.08	(−) 1.99	50.09	0.156	0.145	0.137	0.03
SSR08	0.03	(−) 1.79	55.75	0.212	0.207	0.176	0.072
SSR09	1999	(−) 0.63	158.03	0.726	0.647	0.354	0.173
SSR10	622	(−) 1.95	51.14	0.818	0.789	0.61	0.087
Mean	509.61	1.41	122.9	0.583	0.55	0.464	0.07

The mean expected heterozygosity (Mean *H*_e_), mean observed heterozygosity (Mean *H*_o_) and inbreeding coefficient (F_is_) were averaged across all populations for each loci. Loci ID can be found in Supplementary Table S1 in Supplementary Information. H_o_, H_e_ and F_is_ estimates within population by locus can be found in Supplementary Table S3 in Supplementary Information.

**Table 3:** Geographical and genomic cline analysis of SNPs with narrow cline width (<51 km) selected from a set of 86 SNP markers for *P. glauca, P. engelmannii* and hybrid populations

*SNP_ID*	*Centre*	*Slope*	*Width (km)*	*Type of selection*	*Annotation*	*F*_*st*_
13_496_NS	503.2763	1.99	50.25125628	Directional selection toward Eng	FK506-binding protein	0.29291
288_302_NS	1419.01	−1.994882	50.12827826	Overdominance	Late elongated hypocotyl	0.11774
68_286_S	948.864	−1.999534	50.01165272	No cline	Glycosyl hydrolase	0.4658
69_753_S	445.1634	−1.999811	50.00472545	—	CBL-interacting protein kinase	0.13921
71_365_NS	1999.9	−2	50	No cline	Ubiquitin-conjugating enzyme	0.10587
85_279_S	1320.5997	−1.994239	50.14444106	Directional selection toward white	Annexin	0.11071
SS_CO483349-contig3-358	0.0085	−1.982363	50.44484789	Directional selection toward Eng	Unknown	0.10401

Abbreviation: SNP, single-nucleotide polymorphism.

Detailed results for all SNP loci can be found in Supplementary Table S5.
