# Supplementary material for: Genetic architecture and genomic patterns of gene flow between hybridizing species of Picea
Source: Heredity (Edinb). 2015 Mar 25;115(2):153–64. doi: 10.1038/hdy.2015.19 (PMC4815442; doi:10.1038/hdy.2015.19)
Supplement: Supplementary Information [file hdy201519x1.doc]

**Supplementary Information**

**Table S1 Characteristics of 10 nuclear microsatellites markers used in this study (adapted from Rungis *et al*., 2004). Microsatellite ID is used to identify individual loci in subsequent tables.**

| ID | Code | Primer sequence (5' 3') | SSR Motif | Allele size range | Annealing T(c) |
| --- | --- | --- | --- | --- | --- |
| SSR01 | WS0011.P12 | F:cgataagatggctcctcaaa | (AGGA)_32_ | 291-295 | 62 |
|  |  | R:ggaggctgaaaagtggttaca |  |  |  |
| SSR02 | WS0033.A18 | F:ggctgctctcttatccgtttt | (TA)_26_ | 145-149 | 55 |
|  |  | R:tggctctcatccagaaaagaa |  |  |  |
| SSR03 | WS0046.M11 | F:cactagggcattgggaagaa | (AAG)_6_ | 287 | 60 |
|  |  | R:atgagaggctggggtatgaa |  |  |  |
| SSR04 | WS0061.C21 | F:tttttagcctcatggacgtt | (CTTT)_5_ | 259-279 | 60 |
|  |  | R:ggttaaacggacgctgaaag |  |  |  |
| SSR05 | WS0082.O23 | F:agtgacagttgtcttagcacatca | (TA)_15_ | 214-224 | 60 |
|  |  | R:aaggtttccgatcgcatcta |  |  |  |
| SSR06 | WS0092.A19 | F:tgtggttttctgcttggaaa | (AC)_9_ | 215-223 | 60 |
|  |  | R:cccattttgactttgaaataagc |  |  |  |
| SSR07 | WS0092.M15 | F:gatgttgcaggcattcagag | (TCC)_6_ | 212-218 | 60 |
|  |  | R:gcaccagcatcgattgacta |  |  |  |
| SSR08 | WS0092.H13 | F:ccacgatgtcgttgaaagaa | (GCT)_8_ | 220-226 | 55 |
|  |  | R:tttcagtcttcctgcattcg |  |  |  |
| SSR09 | WS00111.K13 | F:gactgaagatgccgatatgc | (AT)_9_ | 215-225 | 62 |
|  |  | R:ggccatatcatctcaaaataaagaa |  |  |  |
| SSR10 | PAAC17 | F:gaaacaaaaattattacgcg | (AC)_36_ | 132-148 | 53 |
|  |  | R:atgccctcctaatgaatg |  |  |  |

Notes: F= Forward primer; R= Reverse primer

**Table S2. Pairwise population F_st_ (below diagonal) and Nei genetic distance (above diagonal) for 13 populations of *Picea glauca x P. engelmannii* based on ten microsatellites.**

| **Pop** | **E1** | **E2** | **E3** | **CP** | **PG** | **EK** | **BV** | **MR** | **MG** | **QL** | **FNL** | **WK** | **FN** |
| --- | --- | --- | --- | --- | --- | --- | --- | --- | --- | --- | --- | --- | --- |
| **E1** | 0 | 0.132 | 0.220 | 0.135 | 0.120 | 0.136 | 0.124 | 0.153 | 0.141 | 0.143 | 0.120 | 0.113 | **0.262** |
| **E2** | 0.061 | 0 | 0.098 | 0.062 | 0.054 | 0.036 | 0.074 | 0.118 | 0.087 | 0.088 | 0.049 | 0.038 | **0.271** |
| **E3** | 0.079 | 0.043 | 0 | 0.111 | 0.083 | 0.060 | 0.085 | 0.088 | 0.124 | 0.123 | 0.111 | 0.091 | **0.287** |
| **CP** | 0.051 | 0.026 | 0.038 | 0 | 0.017 | 0.034 | 0.021 | 0.057 | 0.029 | 0.031 | 0.020 | 0.025 | 0.127 |
| **PG** | 0.043 | 0.028 | 0.029 | 0.007 | 0 | 0.020 | 0.023 | 0.054 | 0.038 | 0.037 | 0.029 | 0.024 | 0.141 |
| **EK** | 0.051 | 0.018 | 0.023 | 0.012 | 0.007 | 0 | 0.030 | 0.061 | 0.052 | 0.039 | 0.026 | 0.031 | 0.180 |
| **BV** | 0.048 | 0.031 | 0.029 | 0.008 | 0.009 | 0.010 | 0 | 0.044 | 0.029 | 0.031 | 0.030 | 0.033 | 0.116 |
| **MR** | 0.059 | 0.046 | 0.031 | 0.020 | 0.020 | 0.020 | 0.016 | 0 | 0.037 | 0.038 | 0.055 | 0.074 | 0.146 |
| **MG** | 0.051 | 0.033 | 0.039 | 0.009 | 0.014 | 0.016 | 0.010 | 0.013 | 0 | 0.020 | 0.031 | 0.050 | 0.105 |
| **QL** | 0.054 | 0.038 | 0.044 | 0.013 | 0.014 | 0.014 | 0.014 | 0.013 | 0.009 | 0 | 0.024 | 0.057 | 0.106 |
| **FNL** | 0.048 | 0.023 | 0.039 | 0.006 | 0.012 | 0.010 | 0.011 | 0.019 | 0.010 | 0.010 | 0 | 0.033 | 0.122 |
| **WK** | 0.043 | 0.020 | 0.033 | 0.010 | 0.010 | 0.012 | 0.013 | 0.027 | 0.020 | 0.024 | 0.015 | 0 | 0.181 |
| **FN** | **0.092** | **0.088** | **0.095** | 0.042 | 0.049 | 0.057 | 0.042 | 0.053 | 0.037 | 0.038 | 0.037 | 0.062 | 0 |

**Table S3 Genetic diversity estimates based on ten microsatellites loci. Sample size (N), number of alleles (N_a_), number of expected alleles (A_e_), allelic richness (A_r_), observed heterozygosity (H_o_), expected heterozygosity (H_e_), unbiased expected heterozygosity (UH_e_) and inbreeding coefficient (F_is_) in populations of *P.glauca x P.engelmannii***

| Pop | Locus | N | N_a_ | H_o_ | H_e_ | F_is_ |
| --- | --- | --- | --- | --- | --- | --- |
| E1 | SSR01 | 2 | 2 | 1.000 | 0.500 | -1.000 |
|  | SSR02 | 6 | 7 | 0.667 | 0.806 | 0.259 |
|  | SSR03 | 5 | 4 | 0.400 | 0.480 | 0.2727 |
|  | SSR04 | 5 | 2 | 0.000 | 0.480 | 1.000 |
|  | SSR05 | 4 | 4 | 0.250 | 0.656 | 0.7 |
|  | SSR06 | 6 | 2 | 0.333 | 0.278 | -0.111 |
|  | SSR07 | 6 | 3 | 0.667 | 0.486 | -0.290 |
|  | SSR08 | 4 | 2 | 0.250 | 0.219 | -0.143 |
|  | SSR09 | 4 | 3 | 0.250 | 0.594 | 0.66 |
|  | SSR10 | 6 | 5 | 0.500 | 0.736 | 0.4 |
| E2 | SSR01 | 16 | 2 | 0.688 | 0.498 | -0.3525 |
|  | SSR02 | 17 | 8 | 0.882 | 0.851 | -0.0063 |
|  | SSR03 | 20 | 7 | 0.800 | 0.765 | -0.0201 |
|  | SSR04 | 19 | 3 | 0.263 | 0.425 | 0.404 |
|  | SSR05 | 17 | 8 | 0.941 | 0.739 | -0.245 |
|  | SSR06 | 20 | 4 | 0.250 | 0.379 | 0.362 |
|  | SSR07 | 18 | 2 | 0.056 | 0.054 | -0.029 |
|  | SSR08 | 16 | 5 | 0.313 | 0.281 | -0.079 |
|  | SSR09 | 17 | 2 | 0* | 0.111 | 1.000 |
|  | SSR10 | 18 | 8 | 0.444* | 0.826 | 0.483^§^ |
| E3 | SSR01 | 17 | 2 | 0.471 | 0.484 | 0.058 |
|  | SSR02 | 18 | 7 | 0.5* | 0.752 | 0.359 |
|  | SSR03 | 18 | 3 | 0.667 | 0.653 | 0.0073 |
|  | SSR04 | 17 | 2 | 0.176 | 0.251 | 0.3239 |
|  | SSR05 | 17 | 5 | 0.882 | 0.680 | -0.269 |
|  | SSR06 | 19 | 3 | 0.368 | 0.536 | 0.337 |
|  | SSR07 | 16 | 3 | 0.188 | 0.174 | -0.046 |
|  | SSR08 | 17 | 3 | 0.294 | 0.348 | 0.183 |
|  | SSR09 | 15 | 2 | 0* | 0.444 | 1.000^§^ |
|  | SSR10 | 19 | 7 | 0.737 | 0.773 | 0.073 |
| CP | SSR01 | 65 | 2 | 0.307 | 0.499 | 0.389^§^ |
|  | SSR02 | 69 | 9 | 0.667* | 0.808 | 0.1816 |
|  | SSR03 | 67 | 7 | 0.522 | 0.696 | 0.256^§^ |
|  | SSR04 | 66 | 4 | 0.258* | 0.490 | 0.479^§^ |
|  | SSR05 | 65 | 8 | 0.769* | 0.734 | -0.04 |
|  | SSR06 | 69 | 10 | 0.435* | 0.596 | 0.277^§^ |
|  | SSR07 | 68 | 4 | 0.044* | 0.072 | 0.39 |
|  | SSR08 | 62 | 5 | 0.161* | 0.195 | 0.182 |
|  | SSR09 | 55 | 9 | 0.436* | 0.762 | 0.435^§^ |
|  | SSR10 | 69 | 10 | 0.493 | 0.772 | 0.368^§^ |
| PG | SSR01 | 83 | 4 | 0.446 | 0.518 | 0.144 |
|  | SSR02 | 86 | 8 | 0.534* | 0.799 | 0.335^§^ |
|  | SSR03 | 85 | 6 | 0.682 | 0.717 | 0.0548 |
|  | SSR04 | 81 | 6 | 0.395* | 0.437 | 0.103^§^ |
|  | SSR05 | 82 | 10 | 0.732* | 0.777 | 0.0648 |
|  | SSR06 | 86 | 7 | 0.477* | 0.628 | 0.247^§^ |
|  | SSR07 | 82 | 4 | 0.146* | 0.240 | 0.395^§^ |
|  | SSR08 | 79 | 4 | 0.114* | 0.209 | 0.458^§^ |
|  | SSR09 | 63 | 8 | 0.253* | 0.638 | 0.607^§^ |
|  | SSR10 | 85 | 8 | 0.518* | 0.791 | 0.35^§^ |
| EK | SSR01 | 74 | 3 | 0.446 | 0.492 | 0.101 |
|  | SSR02 | 85 | 9 | 0.776* | 0.772 | -0.0005 |
|  | SSR03 | 84 | 8 | 0.785 | 0.748 | -0.0448 |
|  | SSR04 | 70 | 7 | 0.286* | 0.504 | 0.438^§^ |
|  | SSR05 | 73 | 9 | 0.726* | 0.775 | 0.0703 |
|  | SSR06 | 86 | 8 | 0.418* | 0.611 | 0.3207 |
|  | SSR07 | 75 | 4 | 0.093* | 0.161 | 0.426^§^ |
|  | SSR08 | 71 | 5 | 0.155 | 0.184 | 0.163 |
|  | SSR09 | 49 | 9 | 0.204* | 0.488 | 0.588^§^ |
|  | SSR10 | 83 | 8 | 0.675* | 0.828 | 0.19^§^ |
| BV | SSR01 | 56 | 3 | 0.304* | 0.525 | 0.429 |
|  | SSR02 | 68 | 8 | 0.853 | 0.818 | -0.0354 |
|  | SSR03 | 68 | 6 | 0.721 | 0.643 | -0.1142 |
|  | SSR04 | 57 | 3 | 0.263* | 0.389 | 0.331^§^ |
|  | SSR05 | 65 | 9 | 0.831* | 0.730 | -0.1305 |
|  | SSR06 | 67 | 8 | 0.567* | 0.694 | 0.189^§^ |
|  | SSR07 | 63 | 4 | 0.095* | 0.121 | 0.223 |
|  | SSR08 | 55 | 5 | 0.109* | 0.244 | 0.558^§^ |
|  | SSR09 | 42 | 10 | 0.405* | 0.733 | 0.457^§^ |
|  | SSR10 | 69 | 7 | 0.681* | 0.755 | 0.105^§^ |
| MR | SSR01 | 41 | 4 | 0.39* | 0.490 | 0.216^§^ |
|  | SSR02 | 50 | 8 | 0.8* | 0.796 | 0.005 |
|  | SSR03 | 48 | 8 | 0.83* | 0.714 | -0.157 |
|  | SSR04 | 41 | 4 | 0.170 | 0.345 | 0.514 |
|  | SSR05 | 46 | 7 | 0.696* | 0.671 | -0.0256 |
|  | SSR06 | 49 | 8 | 0.428 | 0.628 | 0.3269 |
|  | SSR07 | 44 | 4 | 0.068* | 0.129 | 0.482^§^ |
|  | SSR08 | 43 | 4 | 0.163 | 0.153 | -0.05 |
|  | SSR09 | 35 | 9 | 0.629 | 0.794 | 0.222 |
|  | SSR10 | 45 | 8 | 0.644 | 0.825 | 0.229 |
| MG | SSR01 | 51 | 2 | 0.392 | 0.457 | 0.151 |
|  | SSR02 | 75 | 9 | 0.827* | 0.818 | -0.0038 |
|  | SSR03 | 74 | 8 | 0.811* | 0.715 | -0.127 |
|  | SSR04 | 71 | 8 | 0.324* | 0.517 | 0.379^§^ |
|  | SSR05 | 52 | 9 | 0.673* | 0.680 | 0.0203^§^ |
|  | SSR06 | 61 | 9 | 0.475 | 0.731 | 0.356^§^ |
|  | SSR07 | 65 | 2 | 0.062 | 0.060 | -0.024 |
|  | SSR08 | 69 | 6 | 0.246* | 0.287 | 0.148 |
|  | SSR09 | 44 | 11 | 0.659 | 0.857 | 0.241^§^ |
|  | SSR10 | 71 | 8 | 0.577* | 0.800 | 0.284^§^ |
| QL | SSR01 | 76 | 2 | 0.316 | 0.411 | 0.238 |
|  | SSR02 | 113 | 9 | 0.832 | 0.835 | 0.0082 |
|  | SSR03 | 109 | 8 | 0.716 | 0.748 | 0.0484 |
|  | SSR04 | 89 | 8 | 0.539* | 0.648 | 0.172 |
|  | SSR05 | 83 | 8 | 0.807* | 0.644 | -0.2475 |
|  | SSR06 | 93 | 11 | 0.569* | 0.716 | 0.209^§^ |
|  | SSR07 | 95 | 4 | 0.105 | 0.138 | 0.244 |
|  | SSR08 | 90 | 4 | 0.056 | 0.054 | -0.014 |
|  | SSR09 | 59 | 10 | 0.54* | 0.813 | 0.340^§^ |
|  | SSR10 | 101 | 10 | 0.673 | 0.789 | 0.151^§^ |
| FNL | SSR01 | 63 | 3 | 0.556 | 0.496 | -0.111 |
|  | SSR02 | 75 | 9 | 0.827 | 0.830 | 0.0109 |
|  | SSR03 | 67 | 7 | 0.701 | 0.704 | 0.011 |
|  | SSR04 | 69 | 8 | 0.536 | 0.596 | 0.107 |
|  | SSR05 | 64 | 7 | 0.75* | 0.673 | -0.107^§^ |
|  | SSR06 | 74 | 7 | 0.595 | 0.571 | -0.0343 |
|  | SSR07 | 70 | 4 | 0.043 | 0.042 | -0.007 |
|  | SSR08 | 66 | 5 | 0.121* | 0.184 | 0.348 |
|  | SSR09 | 43 | 10 | 0.372* | 0.725 | 0.495^§^ |
|  | SSR10 | 78 | 9 | 0.744* | 0.830 | 0.11^§^ |
| WK | SSR01 | 27 | 2 | 0.185 | 0.489 | 0.632^§^ |
|  | SSR02 | 41 | 9 | 0.854 | 0.807 | -0.0456 |
|  | SSR03 | 41 | 6 | 0.610 | 0.721 | 0.166 |
|  | SSR04 | 29 | 6 | 0.345 | 0.359 | 0.057 |
|  | SSR05 | 36 | 8 | 0.917* | 0.699 | -0.298^§^ |
|  | SSR06 | 41 | 9 | 0.439* | 0.448 | 0.0316 |
|  | SSR07 | 32 | 4 | 0.219 | 0.202 | -0.069 |
|  | SSR08 | 30 | 3 | 0.133* | 0.184 | 0.2927 |
|  | SSR09 | 25 | 6 | 0.28* | 0.637 | 0.574^§^ |
|  | SSR10 | 39 | 9 | 0.744 | 0.823 | 0.109 |
| FN | SSR01 | 13 | 2 | 0.462 | 0.497 | 0.111 |
|  | SSR02 | 14 | 7 | 0.786 | 0.798 | 0.053 |
|  | SSR03 | 14 | 4 | 0.429 | 0.643 | 0.365^§^ |
|  | SSR04 | 14 | 2 | 0.429 | 0.459 | 0.1034 |
|  | SSR05 | 14 | 4 | 0.571 | 0.518 | -0.0667 |
|  | SSR06 | 14 | 6 | 0.429 | 0.753 | 0.460^§^ |
|  | SSR07 | 14 | 1 | 0.000 | 0.000 | -- |
|  | SSR08 | 6 | 2 | 0.167 | 0.153 | -0.091 |
|  | SSR09 | 7 | 7 | 0.571 | 0.816 | 0.368 |
|  | SSR10 | 14 | 5 | 0.500 | 0.704 | 0.323 |

Notes: N= sample size, N_a_= Number of different alleles, H_o_= Observed heterozygosity= Number of Hets/N, H_e_= Expected heterozygosity= 1-Sum pi^2; Fis=Inbreeding coefficient; where pi is the frequency of the ith allele for the population

* Significant deviation from Hardy-Weinberg equilibrium (P<0.01)

§ Significant deviation of inbreeding coefficient from zero (P<0.01). A positive value indicates heterozygote deficit and a negative value, heterozygote excess.

**Table S4. Genetic diversity estimates based on 86 SNP markers. Mean number of alleles (Na), observed heterozygosity (Ho) and expected heterozygosity (He) are described for each of the populations in the *Picea glauca x P. engelmannii* hybrid zone.**

|  | **Na** | | **Ho** | | **He** | |
| --- | --- | --- | --- | --- | --- | --- |
| **Pop** | **Mean** | **SE** | **Mean** | **SE** | **Mean** | **SE** |
| **FN** | 1.547 | 0.056 | 0.190 | 0.027 | 0.169 | 0.021 |
| **PG** | 1.895 | 0.037 | 0.314 | 0.026 | 0.290 | 0.018 |
| **QL** | 2.000 | 0.000 | 0.305 | 0.020 | 0.306 | 0.018 |
| **MR** | 2.000 | 0.000 | 0.299 | 0.018 | 0.307 | 0.017 |
| **EK** | 1.977 | 0.016 | 0.272 | 0.019 | 0.284 | 0.019 |
| **WK** | 1.977 | 0.016 | 0.289 | 0.020 | 0.286 | 0.018 |
| **E1** | 1.767 | 0.049 | 0.252 | 0.021 | 0.248 | 0.019 |
| **E2** | 1.651 | 0.054 | 0.197 | 0.023 | 0.207 | 0.021 |
| **E3** | 1.640 | 0.057 | 0.181 | 0.022 | 0.180 | 0.019 |
| **Total** | 1.828 | 0.014 | 0.256 | 0.007 | 0.253 | 0.007 |

**Table S5. Results of the geographical and genomic cline analyses for the *Picea glauca x P. engelmanniii* hybrid zone based on 86 SNP loci.**

| SNP_ID | Geographic cline analysis | | | | Genomic cline analysis | | | F_st_ | Annotation |
| --- | --- | --- | --- | --- | --- | --- | --- | --- | --- |
|  | centre (km) | slope | width (km) | shift from ssr centre 562 | P-value | Type of selection | Genotypes |  |  |
| 100_316_NS | 1700,22 | -0,55 | 181,95 | 1138,22 | 0 | underdominance | EE,WE-,WW+ | 0,11 | Ras-related protein |
| 11_348_S | 45,68 | -1,77 | 56,55 | -516,32 | 0 | overdominance | EE,WE+,WW | 0,10 | chalcone synthase |
| 124_495_S | 1442,44 | -1,78 | 56,34 | 880,44 | 0.001 | neutral | EE,WE,WW | 0,05 | auxin efflux carrier-like protein |
| 127_273_S | 418,53 | 0,19 | 524,11 | -143,47 | 0 | directional selection | EE-,WE,WW+ | 0,11 | glutathione S-transferase-like protein |
| 132_78_S | 0,00 | -0,26 | 388,29 | -562,00 | 0 | overdominance | EE,WE+,WW+ | 0,10 | NAM |
| 133_39_S | 72,58 | -1,69 | 59,35 | -489,42 | 0 | directional selection | EE+,WE,WW- | 0,10 | protease inhibitor/seed storage/lip |
| 133_418_S | 0,05 | -1,88 | 53,19 | -561,95 | 0.006 | neutral | EE,WE,WW | 0,10 | protease inhibitor/seed storage/lip |
| 133_553_NS | 432,55 | 1,02 | 98,34 | -129,45 | 0.099 | neutral | EE,WE,WW | 0,10 | protease inhibitor/seed storage/lip |
| 135_122_NS | 0,02 | -1,66 | 60,07 | -561,98 | 0 | underdominance | EE,WE-,WW+ | 0,08 | glycosyltransferase CAZy family GT34 |
| 136_421_S | 0,01 | -1,95 | 51,31 | -561,99 | 0.25 | neutral | EE,WE,WW | 0,10 | ABC transporter |
| 13_496_NS | 503,28 | 1,99 | 50,25 | -58,72 | 0 | directional selection | EE+,WE,WW- | 0,29 | FK506-binding protein |
| 141_349_S | 0,04 | 0,05 | 2222,22 | -561,96 | 0 | overdominance | EE,WE+,WW- | 0,11 | no hits |
| 162_199_S | 0,01 | -1,51 | 66,42 | -561,99 | 0 | underdominance | EE,WE-,WW+ | 0,10 | xyloglucan endotransglucosylase/hydrolase protein 9 |
| 169_375_NS | 1443,82 | 0,18 | 568,18 | 881,82 | 0 | underdominance | EE,WE-,WW+ | 0,10 | peroxidase |
| 191_162_S | 0,03 | 0,04 | 2295,32 | -561,97 | 0.757 | neutral | EE,WE,WW | 0,11 | unusual floral organs |
| 195_356_NS | 903,02 | -1,04 | 96,43 | 341,02 | 0 | overdominance | EE,WE+,WW- | 0,11 | SWAP domain |
| 198_447_S | 1643,72 | 0,07 | 1436,88 | 1081,72 | 0 | epistasis | EE,WE,WW+ | 0,10 | kinesin-like calmodulin-binding |
| 19_567_S | 0,05 | -1,94 | 51,55 | -561,95 | 0 | no cline | -- | 0,10 | auxin responsive |
| 205_292_S | 566,85 | -0,25 | 402,83 | 4,85 | 0 | directional selection | EE+,WE,WW- | 0,10 | LRR-sous groupe2 |
| 206_435_NS | 34,08 | 1,64 | 60,91 | -527,92 | 0 | directional selection | EE+,WE-,WW- | 0,11 | isoflavone reductase |
| 209_523_S | 237,70 | 0,06 | 1795,33 | -324,30 | 0 | overdominance | EE,WE+,WW | 0,10 | histone H2A |
| 20_374_NS | 0,01 | -1,68 | 59,49 | -561,99 | 0.02 | neutral | EE,WE,WW | 0,10 | gigantea |
| 213_153_S | -- | -- | -- | -- | 0 | directional selection | EE-,WE,WW+ | 0,10 | sphingolipid desaturase |
| 213_330_S | 0,01 | -1,77 | 56,51 | -561,99 | 0 | no cline | -- | 0,10 | sphingolipid desaturase |
| 213_468_NS | 1203,40 | 0,06 | 1694,92 | 641,40 | 0 | directional selection | EE-,WE,WW+ | 0,10 | sphingolipid desaturase |
| 214_180_S | 0,00 | -1,76 | 56,77 | -562,00 | 0 | no cline | -- | 0,10 | argonaute/Zwille-like protein |
| 214_558_S | 0,04 | -1,92 | 52,08 | -561,96 | 0 | no cline | -- | 0,10 | argonaute |
| 215_132_S | 177,15 | -1,30 | 76,94 | -384,85 | 0 | directional selection | EE+,WE,WW- | 0,10 | ABA-responsive element binding factor |
| 222_305_S | 0,01 | -1,79 | 55,95 | -561,99 | 0 | no cline | -- | 0,11 | no hits |
| 222_370_S | 0,06 | -1,83 | 54,64 | -561,94 | 0 | directional selection | EE+,WE,WW- | 0,10 | no hits |
| 234_171_S | 0,00 | -1,62 | 61,77 | -562,00 | 0 | overdominance | EE,WE+,WW- | 0,06 | annexin |
| 242_241_S | 0,01 | 0,12 | 842,46 | -561,99 | 0.102 | neutral | EE,WE,WW | 0,10 | hypothetical protein |
| 245_170_NS | 450,00 | 0,20 | 504,80 | -112,00 | 0 | directional selection | EE-,WE,WW+ | 0,10 | hypothetical protein |
| 245_281_S | 112,16 | -1,69 | 59,07 | -449,84 | 0 | directional selection | EE+,WE,WW- | 0,10 | hypothetical protein |
| 245_98_NS | 79,91 | -0,23 | 439,97 | -482,09 | 0 | directional selection | EE-,WE,WW+ | 0,10 | hypothetical protein |
| 249_648_S | 0,00 | -1,04 | 96,23 | -562,00 | 0 | overdominance | EE,WE+,WW- | 0,10 | unknown |
| 252_200_NS | 1701,03 | -1,36 | 73,57 | 1139,03 | 0 | overdominance | EE,WE+,WW | 0,09 | hypothetical protein |
| 273507_S | -- | -- | -- | -- | 0 | directional selection | EE-,WE,WW+ | 0,10 | glutathione peroxidase |
| 27_420_S | 0,00 | -1,68 | 59,60 | -562,00 | 0 | overdominance | EE,WE+,WW- | 0,10 | basic endochitinase-like protein |
| 27_711_S | -- | -- | -- | -- | 0 | directional selection | EE-,WE,WW+ | 0,10 | basic endochitinase-like protein |
| 27_99_S | 1442,70 | -1,75 | 57,08 | 880,70 | 0 | underdominance | EE,WE-,WW+ | 0,10 | basic endochitinase-like protein |
| 288_302_NS | 1419,01 | -1,99 | 50,13 | 857,01 | 0 | overdominance | EE,WE+,WW- | 0,12 | late elongated hypocotyl |
| 288_628_NS | 458,59 | -0,71 | 141,37 | -103,41 | 0 | overdominance | EE,WE+,WW- | 0,11 | late elongated hypocotyl |
| 295_78_S | -- | -- | -- | -- | 0 | overdominance | EE-,WE+,WW+ | 0,19 | NAM |
| 29_177_S | 0,01 | -1,81 | 55,10 | -561,99 | 0 | overdominance | EE,WE+,WW- | 0,10 | late embryogenesis |
| 29_592_S | 0,00 | -1,88 | 53,12 | -562,00 | 0 | directional selection | EE-,WE,WW+ | 0,10 | late embryogenesis |
| 30_423_S | -- | -- | -- | -- | 0 | directional selection | EE-,WE,WW+ | 0,10 | glutathione S-transferase |
| 41_150_NS | 924,17 | -1,67 | 59,79 | 362,17 | 0.02 | neutral | EE,WE,WW | 0,10 | SKP1-interacting-like protein |
| 42_150_NS | 809,90 | 0,13 | 759,30 | 247,90 | 0 | directional selection | EE-,WE,WW+ | 0,13 | expansin-like protein |
| 45_1067_NS | 763,02 | -0,16 | 631,05 | 201,02 | 0 | directional selection | EE-,WE,WW+ | 0,21 | alpha amylase |
| 50_135_S | 0,02 | 0,26 | 378,21 | -561,98 | 0.548 | neutral | EE,WE,WW | 0,04 | aminoacid permease |
| 50_405_S | 916,50 | 0,14 | 729,93 | 354,50 | 0.675 | neutral | EE,WE,WW | 0,10 | aminoacid permease |
| 51_36_S | 0,01 | -1,38 | 72,40 | -561,99 | 0.015 | neutral | EE,WE,WW | 0,10 | auxin efflux carrier |
| 51_409_NS | -- | -- | -- | -- | 0.001 | neutral | EE,WE,WW | 0,10 | auxin efflux carrier |
| 56_206_S | 1355,00 | 0,13 | 776,40 | 793,00 | 0 | directional selection | EE-,WE,WW+ | 0,09 | CONSTANS |
| 5_1408_NS | 0,03 | -1,86 | 53,78 | -561,97 | 0 | overdominance | EE,WE+,WW | 0,10 | glycosyl hydrolase |
| 68_286_S | 948,86 | -2,00 | 50,01 | 386,86 | 0 | no cline | -- | 0,47 | glycosyl hydrolase |
| 71_365_NS | 1999,90 | -2,00 | 50,00 | 1437,90 | 0 | no cline | -- | 0,11 | ubiquitin-conjugating enzyme |
| 84_370_NS | -- | -- | -- | -- | 0 | overdominance | EE-,WE,WW+ | 0,10 | auxin efflux carrier |
| 85_279_S | 1320,60 | -1,99 | 50,14 | 758,60 | 0 | directional selection | EE-,WE,WW+ | 0,11 | annexin |
| 86_438_S | 533,58 | 1,20 | 83,33 | -28,42 | 0.171 | neutral | EE,WE,WW | 0,10 | phenylcoumaran benzylic ether reductase 3 |
| 89_300_NS | 0,05 | -1,93 | 51,79 | -561,95 | 0 | overdominance | EE,WE+,WW | 0,10 | auxin efflux carrier |
| 89_37_NS | 1999,00 | 1,83 | 54,67 | 1437,00 | 0 | overdominance | EE,WE+,WW | 0,10 | auxin efflux carrier |
| SS_CO483349-358 | 0,01 | -1,98 | 50,44 | -561,99 | 0 | directional selection | EE+,WE,WW- | 0,10 | unknown |
| SS_CO483349-496 | 0,01 | -1,87 | 53,53 | -561,99 | 0 | overdominance | EE,WE+,WW | 0,10 | unknown |
| 69_753_S | 445,16 | -2,00 | 50,00 | -116,84 | 0 | -- |  | 0,14 | CBL-interacting protein kinase |

**Table S6. SNP loci with narrow clines and deviations from neutrality also showed associations with different environmental variables in the *Picea glauca x P. engelmannii* hybrid zone**

|  | MAT | | MAP | | SHM | | PAS | |
| --- | --- | --- | --- | --- | --- | --- | --- | --- |
| SNP loci | Fvalue | Pvalue | Fvalue | Pvalue | Fvalue | Pvalue | Fvalue | Pvalue |
| 13_496_NS | 1.713 | 0.18 | 22.7 | <0.0001 | 3.48 | 0.03 | 37.205 | <0.0001 |
| 288_302_NS | 5.65 | 0.003 | 43.43 | <0.0001 | 16.42 | <0.0001 | 48.429 | <0.001 |
| 85_279_S | 0.32 | 0.72 | 4.89 | 0.0077 | 2.068 | 0.1271 | 6.76 | 0.001 |
| SS_CO483349 | 4.26 | 0.005 | 6.44 | 0.00026 | 2.1712 | 0.09 | 8.15 | <0.0001 |

**Figure S1.** Admixture clines along elevation and latitude for 10 SSR and 86 SNP loci in the *Picea glauca x P. engelmannii* hybrid zones. SSR clines point towards a unimodal distribution whereas SNP clines point toward a trimodal distribution with well-differentiated parental and hybrid habitats.

**Figure S2.** Histogram showing the distribution of hybrid indices in each of the *Picea glauca* x *P. engelmannii* populations based on ten microsatellite markers. Hybrid index indicates the fraction of alleles derived from *P. engelmannii* population, where “0” is pure *P. glauca*, and “1” is pure *P. engelmannii*.

**Figure S3.** Scatterplot of isolation by distance for 13 populations within the *P. glauca* x *P. engelmannii* hybrid zone based on F_st_ pairwise estimates using 10 SSR described in this study. Geographical distance (X-axis) is the distance along latitude from Fort Nelson (FN), British Columbia. Elevational distance (X-axis, graph at bottom) is the distance along elevation from Fort Nelson, British Columbia.

**Figure S4**. Migration and its effect on ancestry and interspecific heterozygosity of parental and hybrid individuals - Simulations were done using Stepping stone migration (A and B) versus Migrant pool migration (C and D), at two-time points - 100 generations (A and C) and 3000 generations (B and D). Parental species are represented by green and blue dots, hybrids, in gray.

**Figure S5**. Boxplots describe associations between genotypic classes and environmental variables for each of the SNPs with narrow geographic clines and genomic clines that deviate from neutrality. Environmental variables tested were precipitation as snow (PAS), mean annual temperature (MAT), mean annual precipitation (MAP) and summer-heat moisture index (SSH). Significance tests results can be found in Table S6.
